# Modeling Brain Functional Connectivity Patterns during an Isometric Arm Force Exertion Task at Different Levels of Perceived Exertion: A Graph Theoretical Approach †

**DOI:** 10.3390/brainsci12111575

**Published:** 2022-11-18

**Authors:** Lina Ismail, Waldemar Karwowski, Farzad V. Farahani, Mahjabeen Rahman, Ashraf Alhujailli, Raul Fernandez-Sumano, P. A. Hancock

**Affiliations:** 1 Department of Industrial and Management Engineering, Arab Academy for Science Technology & Maritime Transport, Alexandria 2913, Egypt; 2 Department of Industrial Engineering and Management Systems, University of Central Florida, Orlando, FL 32816, USA; 3 Department of Biostatistics, Johns Hopkins University, Baltimore, MD 21218, USA; 4 Department of Management Science, Yanbu Industrial College, Yanbu 46452, Saudi Arabia; 5 Department of Psychology, University of Central Florida, Orlando, FL 32816, USA

**Keywords:** electroencephalogram, EEG, functional connectivity, force exertion, graph theory, perceived exertion

## Abstract

The perception of physical exertion is the cognitive sensation of work demands associated with voluntary muscular actions. Measurements of exerted force are crucial for avoiding the risk of overexertion and understanding human physical capability. For this purpose, various physiological measures have been used; however, the state-of-the-art in-force exertion evaluation lacks assessments of underlying neurophysiological signals. The current study applied a graph theoretical approach to investigate the topological changes in the functional brain network induced by predefined force exertion levels for twelve female participants during an isometric arm task and rated their perceived physical comfort levels. The functional connectivity under predefined force exertion levels was assessed using the coherence method for 84 anatomical brain regions of interest at the electroencephalogram (EEG) source level. Then, graph measures were calculated to quantify the network topology for two frequency bands. The results showed that high-level force exertions are associated with brain networks characterized by more significant clustering coefficients (6%), greater modularity (5%), higher global efficiency (9%), and less distance synchronization (25%) under alpha coherence. This study on the neurophysiological basis of physical exertions with various force levels suggests that brain regions communicate and cooperate higher when muscle force exertions increase to meet the demands of physically challenging tasks.

## 1. Introduction

Perceived exertion is the cognitive sensation of work demands associated with voluntary muscular actions [1]. Measurements of exerted force help in determining human physical capability, which is crucial for avoiding the risk of overexertion. Studies on how the brain works and perceives an exerted force are very promising in the field of ergonomics. This understanding is not gained by studying the function of brain regions in isolation but by relating to the pattern of interactions between different brain regions. The human brain can be conceptualized as a complex network. The recent confluence of network science, modern network modeling, advanced computation paradigms, and developments in neurophysiological technologies has shed light on new ways of studying complex intracortical interactions. This field of study is commonly known as brain connectivity or the “connectome” [2,3]. Currently, there are three ways to quantify brain connectivity: (1) structural connectivity, which is determined from anatomical brain links; (2) functional connectivity, which is estimated from statistical dependencies between different brain regions; (3) effective connectivity, which reflects causal relations between activated brain areas [4]. Accordingly, several techniques with different properties and capabilities have been developed to study brain connectivity patterns [5,6,7,8,9,10,11,12]. The most commonly used functional connectivity estimators have been recently discussed by Ismail and Karwowski (2020) [13].

Evaluations of communication patterns can contribute to our understanding of information processing and brain functional organization during the execution of a motor task [14]. Predominant frontal-motor coupling in the alpha band and fronto-occipital coupling in the beta band have been found during a suboptimally controlled task [15]. Moderate changes in brain activity in the prefrontal cortex and greater changes in the parietal lobe have been observed with the elevation of exercise intensity [16]. Porter et al. [17] reported an increase in the functional brain network of the frontal region associated with physical and cognitive exertion tasks. High-resistance pedal exertion strengthens intracerebral connections [18]. High coherence values have suggested an increase in functional connectivity during a muscle exertion task [19]. Previous evidence suggests that cortical brain function is influenced by various exercise modes, preferences, intensities, and workloads [20,21,22,23]. Recently, a graph theoretical approach has been applied to characterize changes in functional network efficiency during physical activities [24]. Graph theory is a powerful mathematical tool [25] that provides a conceptual framework for studying neurophysiological data to characterize brain topological organization and explore brain connectivity patterns [26,27,28].

In the graph theory approach, the brain is graphically modeled as a network consisting of nodes and edges [28,29]. On a macroscopic scale, the graph nodes represent anatomical brain regions (either scalp or cortex sources), and the graph edges represent statistical measures of association, such as anatomical, functional, or effective connections [30]. A variety of graph theoretical measures can be calculated to investigate the topological organization of the brain network as a whole (i.e., global measures) or as individual nodes (i.e., local measures) [31,32,33,34,35,36,37].

Global graph theory measures aim to reveal network functional segregation and global integration of information flows within the brain network. Segregation identifies the degree to which a network’s elements form separate clusters and can be defined by calculating the clustering coefficient, modularity, transitivity, or local efficiency [35]. Integration defines the capacity of a network to exchange information and can be defined by calculating the characteristic path length or global efficiency [33,38]. The network modularity should be calculated to determine the integration and segregation between sub-networks [39,40].

Local graph theory measures, such as betweenness, degree, eigenvector centrality, and nodal efficiency [30,41], are commonly used to evaluate nodal centrality and detect network hubs. Watts and Strogatz [42] demonstrated that the nervous system has small-world properties characterized by strong local clustering among network nodes with short path lengths between neighboring nodes [26]. Small-world networks balance the segregation and integration of information [38,43,44]. Studies of functional and effective brain analysis suggest that the brain has small-world properties that ensure high local and global information transfer efficiency with low wiring costs [44,45,46].

Currently, graph measures are primarily applied to display the function of the central nervous system in clinical investigations [47,48,49,50,51,52,53,54,55,56,57,58]. A few studies have investigated brain networks using graph measures during physical activity. For example, a temporary reduction in brain network efficiency was found when participants approached voluntary exhaustion during an incremental treadmill exercise [18]. Additionally, a significant reduction in local functional measures was found in the theta band when task difficulty increased [17].

The present study aimed to investigate the topological changes in functional brain networks induced by predefined levels of physical exertion using electroencephalogram (EEG) source data in a group of healthy female participants. EEG signatures of brain regions were recorded during several isometric force exertion tasks. We employed EEG coherence analysis to examine and visualize functional connectivity patterns at various levels of force exertion for the alpha and beta bands. We then applied a graph theoretical framework to compare the global measures between various levels of exertion in the alpha and beta bands.

It should be noted here that EEG task-based studies have been previously performed at the scalp level, creating spurious connectivity patterns due to volume-conduction phenomena associated with EEG signals [59]. Most previous studies ignored the quantification of local measures, leading to a loss of balance for the most efficacious type of brain connectivity measures [38]. Considering the limitations of previous studies, we studied functional connectivity patterns in EEG data at the source level using coherence analysis methods for female participants only [60,61]. In this work, we consider sex as a factor, as previous findings have suggested sex and hormone-based differences in brain networks when comparing male and female individuals [62,63] and due to the limited number of EEG-based studies on female participants only [13,64]. Furthermore, recent studies support “the importance of considering sex as a biological variable in brain research” [65] for the future development of sex-specific models to ascribe cognitive functional significance [66] and an understanding of sex-related diseases [67]. We also calculated the most commonly used global and local graph theory measures for two frequency bands (alpha and beta). Based on the study objectives, the following research questions were posed: Are functional connectivity patterns altered by different force exertion levels? Are topological network properties affected by specific force exertion levels?

This paper is organized as follows: Section 2 outlines the methods and procedures, and Section 3 describes the statistical analyses. Section 4 describes the results. Section 5 provides a discussion, and Section 6 discusses study limitations and future implications. In the final section, we present our conclusions.

This study was based on a Ph.D. dissertation titled “Topological Changes in the Functional Brain Networks Induced by Isometric Force Exertions Using a Graph Theoretical Approach: An EEG-based Neuroergonomics Study” [68].

## 2. Materials and Methods

In this section, a pipeline for EEG data pre-processing and processing, coherence estimation, and graph theory measures are described.

### 2.1. Methodological Pipeline

**Figure 1:**
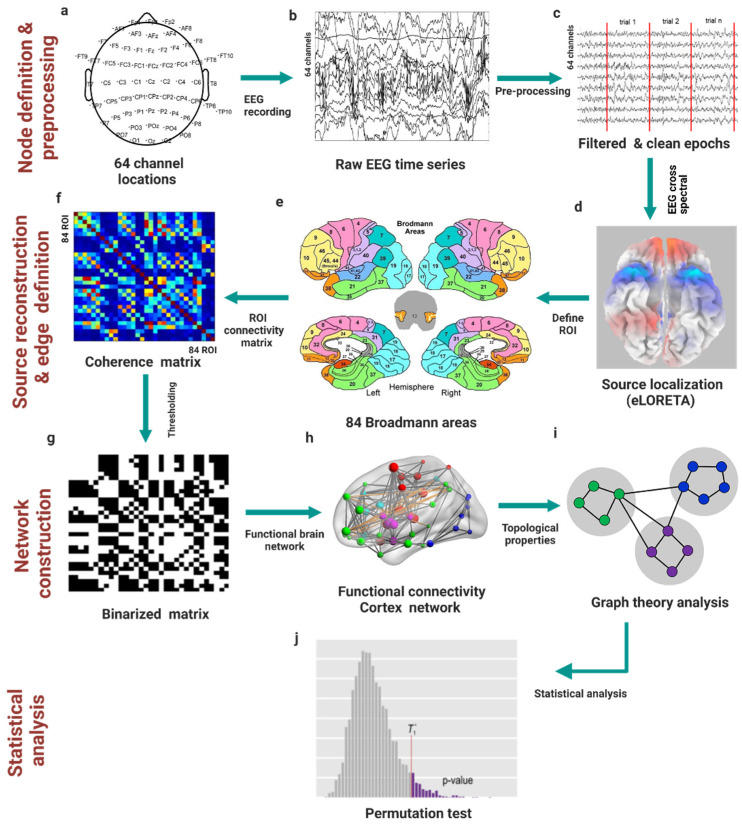
Methodological pipeline. (**a**) Collecting electroencephalogram (EEG) data using 64 channel locations. (**b**) Recording the EEG time series. (**c**) Filtering, cleaning, and epoching the EEG time-series signals. (**d**) Reconstructing the EEG source from the EEG cross spectra using exact low-resolution brain electromagnetic tomography (eLORETA) software. (**e**) Parcellating the cortex according to the Brodmann area (BA) atlas. (**f**) Constructing the adjacent matrix after estimating the connectivity patterns. (**g**) Binarizing the network using a threshold value. (**h**) Constructing the functional connectivity patterns between regions of interest (ROIs). (**i**) Calculating graph theoretical measures to compute local and global network properties. (**j**) Applying a nonparametric permutation test to assess brain topological changes.

An overview of the novel methodological pipeline proposed in this work is shown in Figure 1. First, EEG data were collected for all participants using 64 channel locations to define the network nodes. Then, the collected EEG time-series signals underwent pre-processing. A fast Fourier transform algorithm using Hanning windows was used to calculate cross spectra for each frequency band for each participant’s exertion level for the cleaned and filtered EEG epochs. Using the exact low-resolution brain electromagnetic tomography (eLORETA) transformation matrix, we transformed the cross spectra for each subject and frequency band to eLORETA files [69,70], and we reconstructed the EEG source. The solution space estimated by source localization was then parcellated into brain anatomical structures according to the Brodmann atlas, which was used to define 84 Brodmann area (BA) regions of interest (ROIs) (i.e., graph nodes) for brain network construction. Functional connectivity was estimated across all pairs of brain regions. This step yielded an adjacent matrix (sized 84 × 84) for each participant and EEG frequency band at each force exertion level, which was binarized to remove weak connections [71]. Graph theoretical measures were then used to compute the local and global network properties. Finally, statistical analyses based on nonparametric permutation tests were applied to assess brain topological changes under experimental conditions.

### 2.2. Participant Selection and Ethical Code

Twelve healthy adult female participants (mean age 28 ± 6 y) performed a physical task of isometric arm exertion. All participants met the experimental requirements, including the absence of fatigue-related or chronic physical disorders, musculoskeletal disease, back pain, injuries, mental illness, history of cardiovascular problems, and neurological disorders. Pregnant female participants were excluded from the study. All participants were instructed not to consume any medication, coffee, or alcohol for 24 h and not to engage in any exercise for 48 h prior to the experiment. Experiments were conducted in a temperature-controlled and sound-attenuated environment in the computational neuroergonomics laboratory at the University of Central Florida to control for possible confounds in our study. All experiments were carried out with the approval of the Institutional Review Board at the University of Central Florida (STUDY00000535). All participants signed an informed consent form after the experimental protocol had been explained by a researcher, and the collected data were treated confidentially.

### 2.3. Apparatus

The isometric arm exertion task was performed using a Jackson Strength Evaluation System [72]. The experimental protocol was recommended by Chaffin et al. [73]. Participants were asked to exert a force by pulling a chain upward using their flexed arms without any body movement [73,74]. A TORBAL FC 5k series force measurement device was attached to the handle to record the exerted arm forces.

### 2.4. Experimental Design

**Figure 2:**
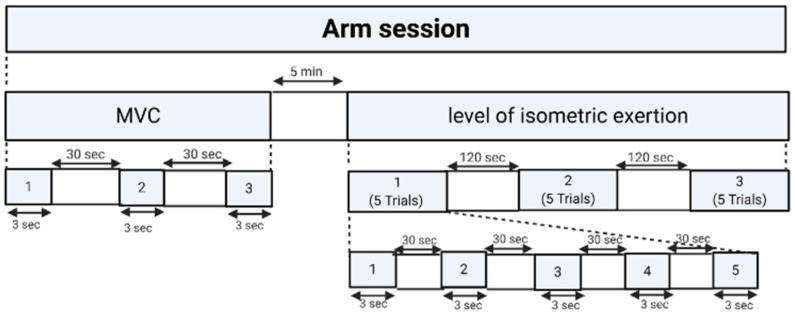
The study protocol for arm force exertion task. MVC: maximum voluntary contraction.

The laboratory experiment was designed to record EEG signals during an isometric arm exertion task at predefined levels of exertion. The participants were instructed to avoid any unnecessary movement during the experiment. The detailed timeline for the experimental sessions is shown in Figure 2.

**Figure 3:**
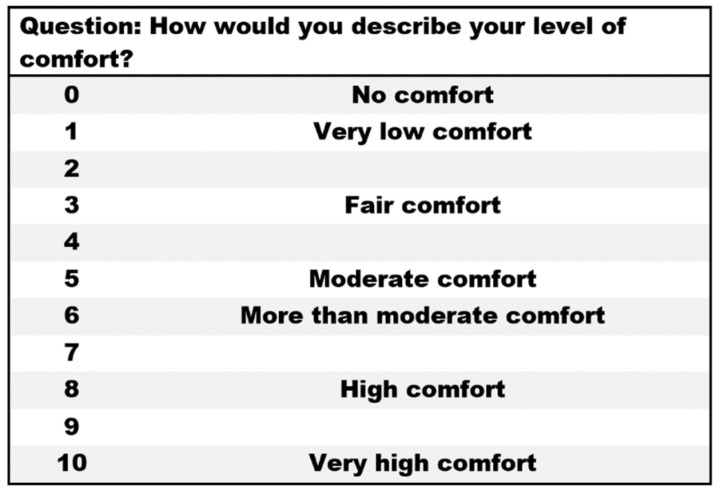
The eleven-point scale of perceived physical comfort [78].

The experiment consisted of two tasks: (1) maximum voluntary contraction (MVC) and (2) isometric force exertion. In the MVC task, the participants were asked to apply the maximum force for 3 s for each of the three trials, with a 30 s rest period between each trial. Then, a 5 min rest was provided to avoid muscle fatigue. In the isometric arm flexion task, participants were asked to exert a force that matched one of five predefined exertion levels: (1) extremely light, (2) light, (3) somewhat hard, (4) hard, and (5) extremely hard. These predefined force levels were adapted from a 6–20 scale of the rate of perceived exertion (RPE) proposed by Borg [75], a well-known and validated scale commonly used in ergonomics research [76,77]. The participants were asked to maintain steady-state exertion for 3 s for three trials, with a 120 s rest period between each trial. After each trial, the participants were asked to subjectively assess the level of physical comfort that corresponded to the exerted force (N) using an 11-point scale of perceived physical comfort proposed by Karwowski, W. [78] (see Figure 3). The order of force exertion levels was randomly determined to prevent potential learning effects.

### 2.5. EEG Data Acquisition

**Figure 4:**
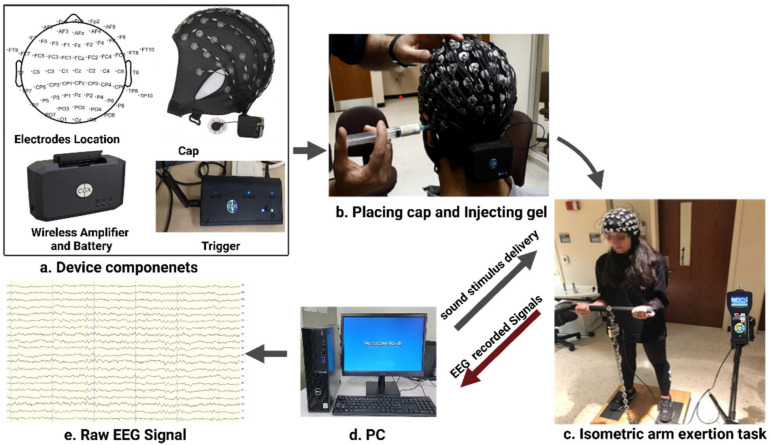
EEG data acquisition system and its components. (**a**) Device components. (**b**) Placing cap and injecting gel. (**c**) Isometric arm exertion task. (**d**) PC. (**e**) Raw EEG Signal.

For the experimental setup, we have followed the recommended procedure addressed in previous studies [79,80]. We used a gel-based EEG wireless system composed of 64 EEG channels and an elastic electrode cap in different sizes with active Ag/AgCl electrodes positioned according to the 10–10 international montage system, trigger, and wireless amplifier (Figure 4a). We began with a manual abrasion of the participants’ scalps with a hard-bristle comb for removing dead skin. Participants were asked to wash their hair without additives such as hair styling products or conditioners to avoid greasing layers. Then, we measured the distance between the participants’ nasion and inion to ensure that the Cz electrode was placed at the center of the head, followed by measuring the nasion to the Cz to ensure that the distance was half of the distance from nasion to inion. We mounted the cap on the participant’s head and tightened the cap with a chin strap. Then, we turned on the EEG device and checked the wireless connection. A cotton swab with isopropyl alcohol was used to clean the skin for each electrode opening in the cap.

Then we filled the electrode cap opening by injecting superVisc electrolyte gel with a small syringe to reduce impedance and interference (Figure 4b), which will increase the connectivity between the scalp and electrodes. Applying too much gel may create a bridge between the signals of neighboring electrodes. The impedance was kept below 10 kΩ with continuous monitoring throughout the experiment. Physiological signals were sampled at 500 Hz with a bandpass filter of 0.1–100 Hz to avoid anti-aliasing. The participant starts to exert the required forced (Figure 4c), as heard from the stimulus generation software using E-prime 3.0 software [81], which sends markers to the computer (Figure 4d). The collected raw EEG signals (Figure 4d) were acquired using Cognionics acquisition software [82].

### 2.6. EEG Pipeline Analysis

**Figure 5:**
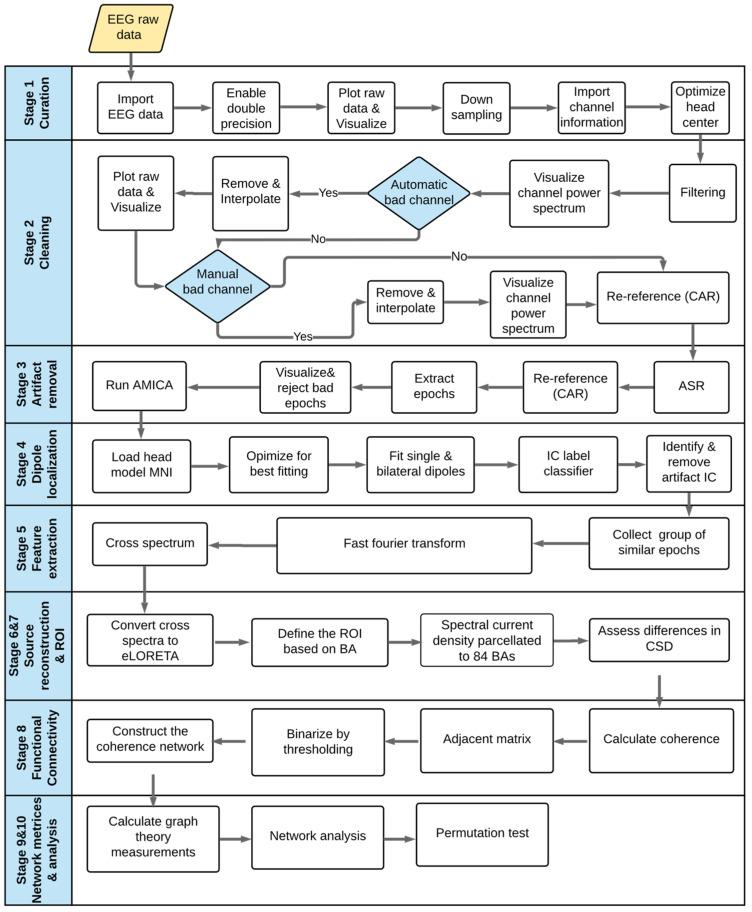
The data processing workflow. AMICA: adaptive mixture independent-component analysis, ASR: artifact substance reconstruction, BA: Brodmann area, CAR: common average referencing, CSD: current source density, eLORETA: exact low-resolution brain electromagnetic tomography, IC: independent component, MNI: Montreal Neurological Institute, and ROI: region of interest.

The data processing workflow consisted of ten stages (see Figure 5), including data curation, cleaning, artifact removal, dipole localization, feature extraction, source reconstruction, ROI definition, functional connectivity estimation, graph theory calculations, and statistical analyses.

The first four stages primarily relate to data pre-processing, followed by five main steps for processing. Because raw EEG data are most likely contaminated with artifacts, filtering, denoising, and cleaning are crucial for enhancing the signal-to-noise ratio [83,84,85]. EEG pre-processing was performed using EEGLAB (version 14.1.2 [86], an open-source toolbox that runs on MATLAB R2019b software (MathWorks, Natick, MA, USA)).

Curation (Stage 1): The raw EEG data were imported, ensuring a double-precision option [87]. The data were visually inspected, and the sample was reduced from 500 to 250 Hz for easier storage and faster processing. The Montreal Neurological Institute (MNI) coordinates were used for defining channel locations, and the head center was optimized to fit the head sphere.

Cleaning (Stage 2): EEG signals were filtered through a 1–50 Hz zero-phase Hamming window known as a finite impulse response bandpass filter [88,89,90,91]. The spectra for the 64 channels were plotted and manually visualized. Then, an automatic bad channel rejection process was applied using the EEGLAB toolbox known as clean_raw data [92]. This automatic toolbox can detect and separate noisy channels and low-frequency drifts. Interpolation was applied after bad channels had been detected and removed to alleviate the bias resulting from an unequal number of electrodes between the right- and left-brain hemispheres. An average re-reference procedure was applied to reset the data to zero-sum across channels.

Artifact removal (Stage 3): An artifact substance reconstruction method that subspaces unusually large-amplitude data was applied first for artifact removal and correction. This method does not consider eye-blinking or small-amplitude contamination [93,94]. Consequently, an independent-component analysis (ICA) decomposition method based on the Blind Source Separation technique was used. Motor Imagery Classification Based on Sorted Blind Source Separation, Continuous Wavelet Transform, and Convolutional Neural Network could be used [83]. Before ICA was applied, the continuous EEG data were epoched based on the task structure. For each participant, three MVCs and five isometric exertion levels were repeated three times, resulting in a total of 18 epochs. Thus, for twelve participants, there was a total of 216 epochs. An adaptive mixture ICA (AMICA) algorithm was used to decompose EEG signals into independent components (ICs) [95]. AMICA has been shown to outperform all other ICA approaches [96,97]. An automated classifier known as IC Label was used to distinguish between the brain and non-brain sources [98,99]. AMICA software was retrieved from https://sccn.ucsd.edu/~jason/amica_web.html accessed 10 December 2020.

Dipole localization (Stage 4): Before any ICs were rejected, the sources were localized to separate IC components. An equivalent current dipole and bilateral model were computed for each IC using a boundary element head model [100,101] based on MNI coordinates. DIPFIT version 3, an EEGLAB plugin (Oostenveld and Oostendorp, 2002), was used to calculate the dipole localization. A nonlinear optimization technique using the MATLAB optimizer toolbox was used to locate the best position for a single or bilateral dipole [102]. Residual variances were kept below 40%.

Components that appear to be eye movements or artifacts from blinking, electrocardiography, motion, lines, or noise channels were manually removed after the dipole had been localized. Following the protocol of Nguyen et al. [103], the entire experiment would be rejected if the number of rejected ICs was more than 50% of the total ICs.

Feature extraction (Stage 5): EEG cross spectra were extracted based on a fast Fourier transform using Hanning windows with a 10% onset. The cross spectra were averaged across 50% overlapping windows considering two frequency bands (alpha = 8–13 Hz; beta = 13–30 Hz) for each participant using eLORETA software (freely available at http://www.uzh.ch/keyinst/loreta.htm, accessed on 21 February 2021).

Source reconstruction (Stage 6): Cross spectra for each participant and each frequency band were transformed into eLORETA files using the eLORETA transformation matrix. This resulted in the three-dimensional intracerebral current source density of the electrical neuronal generators for each participant [104]. eLORETA is a genuine inverse solution with exact zero-error localization in the presence of measurements and structured biological noise [69,70,105]. The software uses a realistic head model [106] based on the MNI 152 template [107], with the three-dimensional solution space restricted to the cortical gray matter and hippocampi, as determined by the probabilistic Talairach atlas [108]. The software helps to solve the inverse solution by parcellating the spectral current density into 6,239 voxels with a spatial resolution of 5 mm^3^. eLORETA has been used extensively and has been validated in several studies using real human data [109,110,111,112,113,114,115]. eLORETA helps to determine the distribution of current density across voxels in the brain [116] and has been demonstrated to be more robust and accurate than other source localization methods [37,117].

ROI determination (Stage 7): The cortex was parcellated into ROIs based on anatomical labels corresponding to BAs provided by eLORETA, according to the Talairach Daemon (http://www.talairach.org/, accessed on 1 February 2021) (Supplementary Material A).

Source functional connectivity estimation (Stage 8): EEG source-based functional connectivity matrices were computed using eLORETA software via the coherence method to estimate patterns of statistical dependencies among 84 ROIs for two EEG frequency bands (alpha and beta). The 84 × 84 coherence connectivity matrices were converted to a binary matrix using a set of sparsity threshold ranges to maintain strong connections [71]. Wide sparsity values in the range of 5–50% with steps of 5% were used to prevent the formation of a disconnected network and maintain network reachability.

Network matrices and analysis (Stages 9 and 10): Global and local graph measures were computed for all force exertion levels for two EEG frequency bands. Finally, the network properties were assessed using a nonparametric permutation-based statistical method [118].

### 2.7. Estimation of Functional Connectivity of EEG Cortical Sources

Coherence is a parameter that is widely used to study the functional brain network [15,119,120,121,122,123,124] and is reliable for evaluating physiological abnormalities [125,126]. Coherence refers to the degree of association between two brain regions. According to Walter [127], coherence reflects the phase synchrony of EEG signals recorded between pairs of electrodes in the frequency domain. Mathematically, coherence is defined as the absolute value of the cross spectra of two signals normalized by the spectral power between two signal x and y [6], as shown in Equation (1):

(1) $$\;C_{xy}\left(f\right)=\frac{{\left|W_{xy}\left(f\right)\right|}^{2}}{W_{x}\left(f\right)*\;W_{y}\left(f\right)}$$

where *f* is the frequency, *W_x_* is the power spectral density (or simply power spectrum) of signal *x* at frequency *f*, *W_y_* is the power spectral density (or simply power spectrum of signal *y* at frequency *f,* and $W_{xy}\left(f\right)$ is the cross-spectrum between two signals x and y at frequency *f,*. In this study, the coherence was computed for 84 ROIs using the eLORETA connectivity algorithm [128] for each subject and EEG frequency band (alpha and beta).

### 2.8. Graph Analysis and Computation of Measures

The most common global and local graph measures for each exertion level and frequency band across the network densities (ranging from 0.1 to 0.5, with a step size of 0.05) were calculated.

Global graph measures included the average clustering coefficient, characteristic path length, global efficiency, and small-worldness. The clustering coefficient is a measure of the degree to which nodes in a graph tend to cluster together. The characteristic path length is the average of the shortest route between all pairs of nodes in the network and measures the ability of the network to transfer serial information [129]. *Global efficiency* is the inverse of the characteristic path length, which measures the network’s ability to transfer parallel information [130]. The *small-worldness* is the ratio of the clustering coefficient to the characteristic path length. A small-worldness index greater than 1 indicates a small-world organization of the brain network [42]. The local efficiency measures the efficiency of information transfer limited to neighboring nodes. Modularity is the ability of a graph to be subdivided into modules that are maximally connected within a module and sparsely connected between modules [39].

Local graph theory measures provide information regarding the properties of individual nodes, including degree and betweenness centrality and nodal efficiency [131]. These measures can be used to quantify the relative importance of a node within the overall network [44]. *Degree centrality* is the number of edges connecting a node to all other nodes. The greater the degree of centrality, the more important the node is in the network. Betweenness centrality quantifies the number of times that a node acts as a bridge along the shortest path between two other nodes [132]. *Betweenness centrality* helps in identifying the most central nodes in a network. *Nodal efficiency* measures the ability of information propagation between a node and the remaining nodes in the network [133]. A node with high centrality is known as a network hub [134] and could be classified into different groups of a connector or provincial [131].

## 3. Statistical Analyses

This section presents statistical analyses for the isometric force exerted, rate of perceived comfort, coherence estimated, and graph theory measures at predefined physical exertion levels.

### 3.1. Isometric Force

An analysis of variance (ANOVA) was used to assess the effect of predefined physical exertion levels on the generated arm forces. Tukey’s post hoc multiple-comparison test was also performed to identify significant differences in exerted forces.

### 3.2. Rate of Perceived Physical Comfort

To assess the effect of predefined physical exertion levels on the rate of perceiving physical comfort (RPPC) scores, an ANOVA was used. Tukey’s post hoc multiple-comparison test was also performed to identify significant differences in the RPPC.

### 3.3. Source Functional Connectivity Estimations

For functional connectivity analysis, we utilized a method that applies a single voxel at the centroid of each BA using eLORETA software [109,110,135,136]. A connectivity analysis between pairs of 84 ROIs in two EEG frequency bands was conducted for all physical exertion levels using independent sample t-tests that were corrected for multiple comparisons using a nonparametric randomization method based on the “maximal statistic.” The same permutation test was applied with 5,000 randomizations to identify the critical probability thresholds at significant levels and to correct type 1 errors.

### 3.4. Brain Network Analysis

For graph theoretical measures, a nonparametric permutation test [137] was used to find significance and compare the topological brain properties for predefined force exertion levels, namely: (1) extremely light, (2) light, (3) somewhat hard, (4) hard, and (5) extremely hard. Briefly, for each network measure, we first calculated the between-group differences in the mean values. An empirical distribution of the differences was then obtained by randomly reallocating all values into two predefined force exertion levels and recalculating the mean differences between the two randomized groups (30,000 permutations). The 95th percentile points of the empirical distribution were used as critical values in a one-tailed test to determine whether the observed group differences could occur by chance. For comparisons of nodal measures, Bonferroni correction procedures were used to correct for multiple comparisons [138].

## 4. Results

This section provides results for anthropometric, isometric force, and physical comfort data, functional connectivity patterns, and graph topological measures for the global and local network for all predefined exertion levels.

### 4.1. Anthropometric Characteristics

A summary of anthropometric measurements and static arm flexion strength for all participants is given in Table 1.

### 4.2. Isometric Arm Forces

Descriptive statistics across all subjects (N = 12) for isometric arm forces at various levels of predefined physical exertion are displayed in Table 2. We calculated the percentage of MVC to normalize the forces exerted by each participant, a commonly accepted approach utilized in ergonomics studies [139,140]. ANOVA results regarding the effect of exertion level on the generated arm force (N) are summarized in Table 3, whereas Tukey pairwise comparisons of forces for various exertion levels at the 95% confidence level are shown in Table 4.

**Figure 6:**
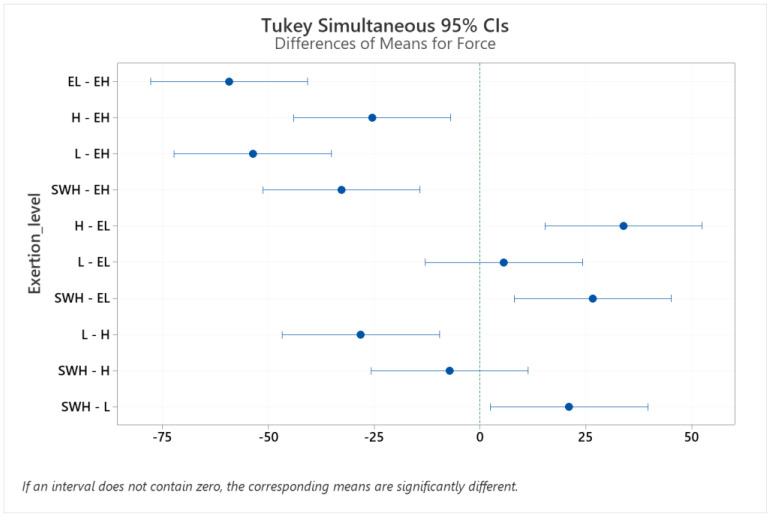
Pairwise comparison between isometric forces at different levels of physical exertion to extremely light (EL), light (L), somewhat hard (SWH), hard (H), and extremely hard (EH). CI: confidence interval.

Pairwise comparisons among exertion levels (extremely hard, hard, somewhat hard, light, and extremely light) were performed using Tukey’s post hoc test, and adjusted *p*-values were computed (see Table 5). Results revealed no significant differences for hard vs. somewhat hard or light vs. extremely light exertion (Figure 6).

### 4.3. Rate of Perceived Physical Comfort

Descriptive statistics across all subjects (N = 12) for RPPC scores at predefined levels of physical exertion are displayed in (Table 6).

The ANOVA results for the effect of exertion level on the RPPC scores are summarized in Table 7. A Tukey pairwise comparison of perceived comfort for various exertion levels at the 95% confidence level is shown in Table 8.

**Figure 7:**
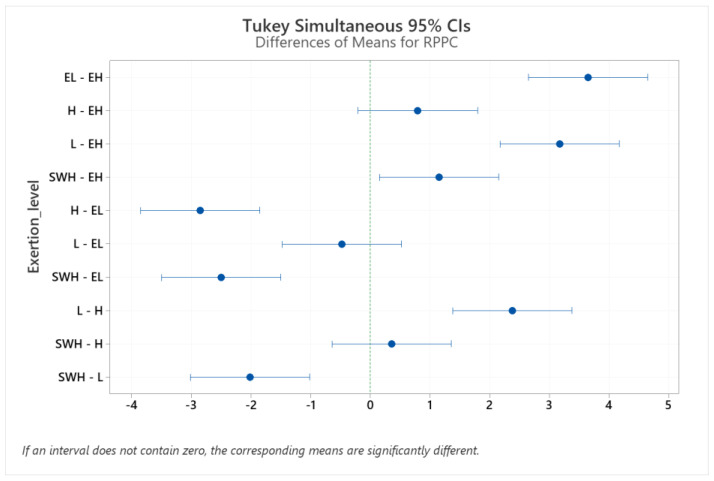
Pairwise comparison between isometric forces at different physical exertion levels to extremely light (EL), light (L), somewhat hard (SWH), hard (H), and extremely hard (EH). CI: confidence interval.

There were no significant differences in the perceived comfort ratings between the exertions of hard and extremely hard, somewhat hard and hard, or extremely light and light (Table 9 and Figure 7).

### 4.4. Functional Connectivity

#### 4.4.1. Functional Brain Patterns

**Figure 8:**
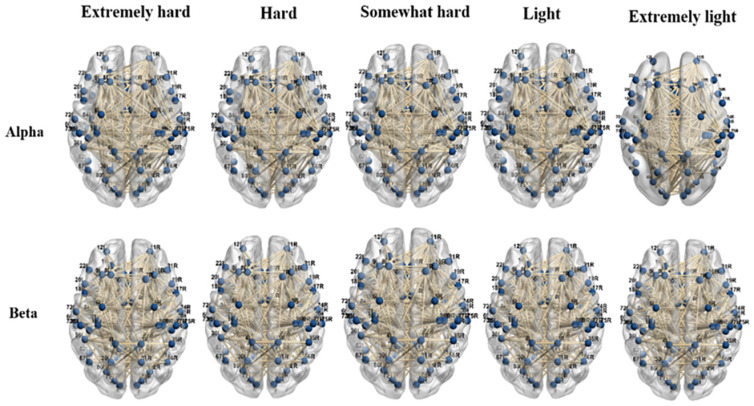
Visualization of the alpha and beta functional brain networks for all exertion levels based on the coherence method.

Coherence matrices were computed for 84 ROIs using the eLORETA connectivity algorithm for each subject and frequency band (alpha and beta) [128]. An overview of the functional brain network for each force exertion level, determined via the coherence method in the alpha and beta frequency bands (research question 1), is provided in Figure 8. The functional interactions between neighboring and distant brain regions were visualized using BrainNet Viewer (http://www.nitrc.org/projects/bnv/, accessed 3 April 2021), a MATLAB toolbox [141]. Overall, the beta coherence networks were found to have more connections in the frontal and temporal lobes than the alpha coherence networks at all force exertion levels, including the left superior frontal gyrus (BA 10), left precentral gyrus (BA 44), right precentral gyrus (BA 44), left inferior frontal gyrus (BA 45), middle frontal gyrus (BA 46), middle temporal gyrus (BAs 21 and 39), left fusiform gyrus (BA 37), and left transverse temporal gyrus (BA 42).

For the alpha band, strong coupling occurs between the left paracentral (BA 5) and the left lingual gyrus (BA 17) and between the left superior frontal gyrus (BA 10) and the middle frontal gyrus (BA 11) when the exertion level increases. Disconnections were found across the middle frontal gyrus (BA 14) and the anterior cingulate (BA 33).

For the beta band, strong coupling occurs between the right superior frontal gyrus (BA 10) and the parahippocampal gyrus (BA 34) when the exertion level increases.

#### 4.4.2. Multiple Comparisons of Functional Connectivity

**Figure 9:**
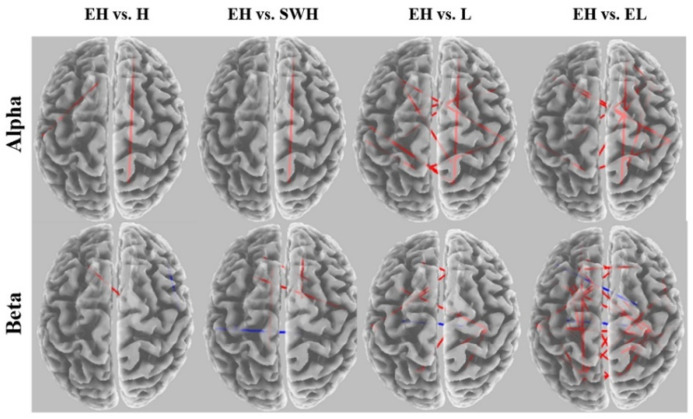
eLORETA wire diagram comparing extremely hard exertion with other exertion levels for each frequency band. EL: extremely light, L: light, SWH: somewhat hard, H: hard, and EH: extremely hard.

For multiple comparisons between different exertion levels, eLORETA software [69] was used to develop wire diagrams. The significant connected regions are mapped in red lines, and the significant disconnected regions are mapped in blue lines. Comparisons of connectivity between the extremely hard exertion level and all other exertion levels, including hard, somewhat hard, light, and extremely light, for each frequency band are shown in Figure 9. We found a significant increase in alpha coherence when the extremely hard exertion is compared with other exertion levels. Disconnections between the left and right hemispheres in the beta network are also present.

**Figure 10:**
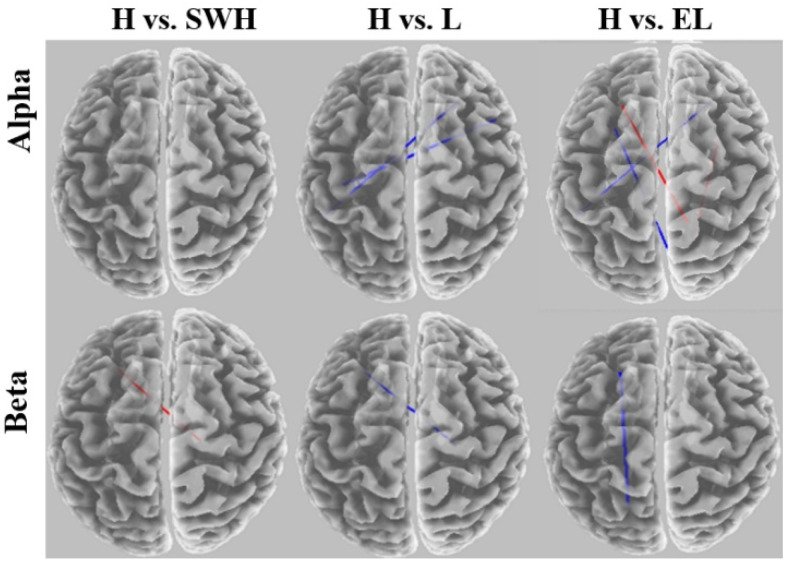
eLORETA wire diagram comparing hard exertion with other exertion levels for each frequency band. EL: extremely light, L: light, SWH: somewhat hard, and H: hard.

Comparisons of connectivity between the hard exertion level and the somewhat hard, light, and extremely light levels for each frequency band are shown in Figure 10. The alpha coherence network was significantly weaker for the hard exertion level than the other exertion levels. No significant differences were observed between the hard and somewhat hard exertion levels. The beta band network showed a significantly greater functional brain network for hard exertion than for the somewhat hard level. In contrast, significant disconnections were found when comparing the light and extremely light exertion levels.

**Figure 11:**
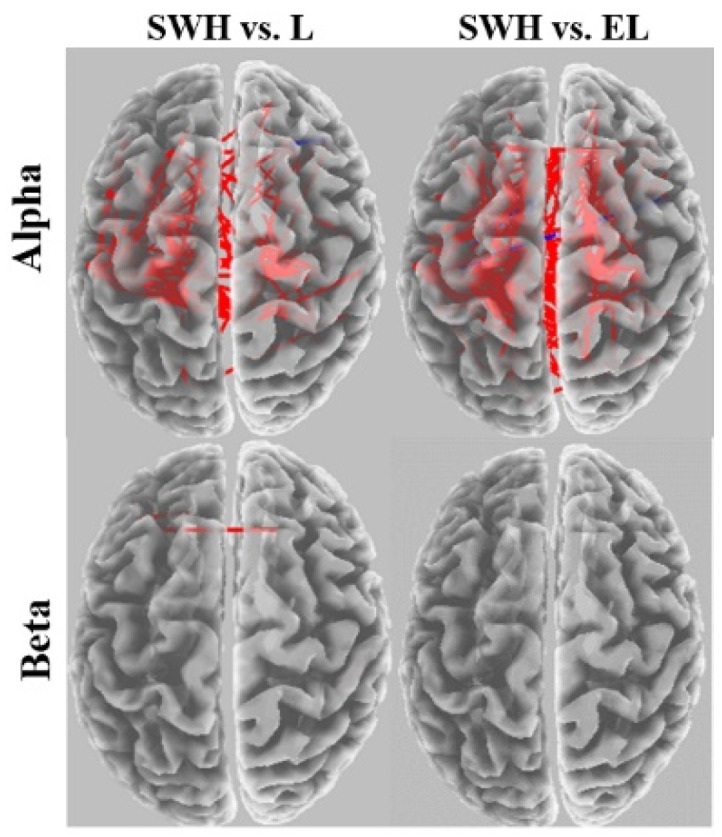
eLORETA wire diagram comparing somewhat hard exertion with other exertion levels for each frequency band. EL: extremely light, L: light, and SWH: somewhat hard.

Comparisons of connectivity between the somewhat hard vs. light and vs. extremely light exertion levels for each frequency band are shown in Figure 11. For the somewhat hard exertion level, the alpha network was found to have denser connections in the frontocentral brain region than networks for the light and extremely light exertion levels. No significant alterations were found in the beta coherence network between somewhat hard and extremely light exertion.

**Figure 12:**
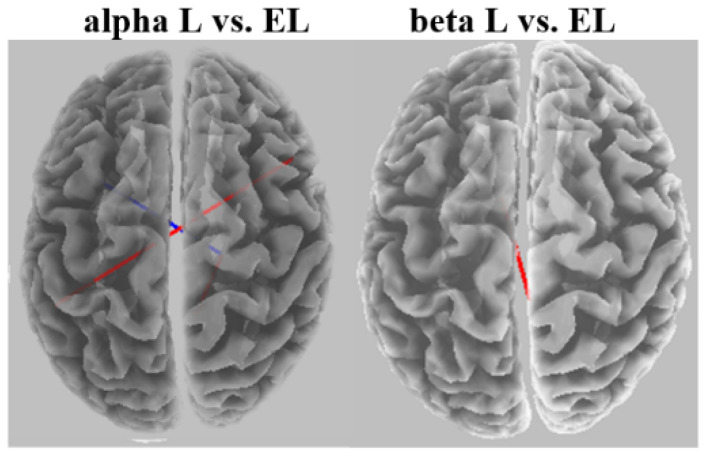
eLORETA wire diagram comparing light with extremely light exertion for each frequency band. EL: extremely light and L: light.

Figure 12 shows a comparison of connectivity between extremely light and light exertion for each frequency band. Significant increases in the coherence connectivity for some cortical regions were found for the alpha and beta networks.

### 4.5. Brain Network Results

This section discusses the topological differences between global and local network measures (research question 2). Global measures were computed using the Brain Connectivity Toolbox (http://www.brain-connectivity-toolbox.net, accessed on 14 April 2021) [34] (http://www.nitrc.org/projects/gretna/, accessed 17 April 2021), while local measures were computed based on a developed Python code.

#### 4.5.1. Topological Differences in the Global Network for Alpha Coherence Network

**Figure 13:**
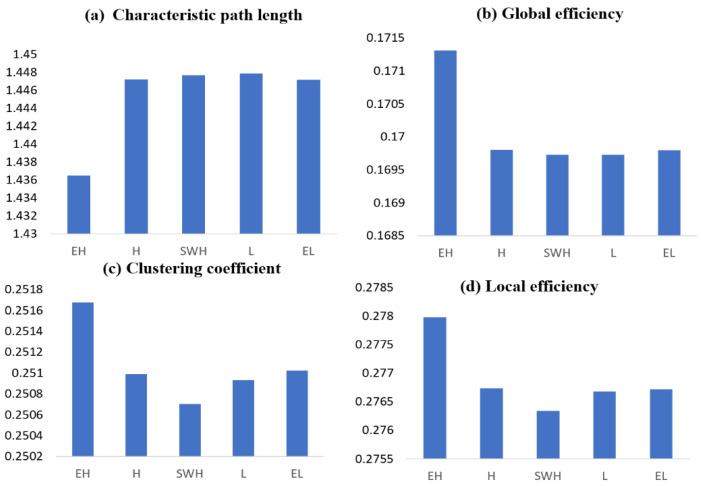
Graph theoretical network metrics showing the main effects of different exertion levels. (**a**) Characteristic path length. (**b**) Global efficiency. (**c**) Clustering coefficient. (**d**) Local efficiency for alpha coherence. EL: extremely light, L: light, SWH: somewhat hard, H: hard, and EH: extremely hard.

A network with a higher small-worldness value was observed for the extremely hard exertion level in the alpha coherence network compared with the other exertion levels (permutation test, *p* < 0.05). Significant changes in the characteristic path length were found for the various exertion levels, with a significant reduction in characteristic path length for hard vs. somewhat hard (permutation test, *p* < 0.0089), hard vs. light (permutation test, *p* < 0.0233), and hard vs. extremely light (permutation test, *p* < 0.0179) (Figure 13a).

A significant increase in the network’s global efficiency was also observed for high exertion levels (see Figure 13b). Significant changes in the clustering coefficient were found for the various exertion levels (Figure 13c). A significant increase was observed between extremely hard and hard (permutation test, *p* < 0.0123), hard and somewhat hard (permutation test, *p* < 0.0353), and hard and extremely light exertion levels (permutation test, *p* < 0.032). A significant reduction was observed between the somewhat hard and light exertion levels (permutation test, *p* < 0.0209). Significant increases in network local efficiency were found for extremely hard vs. hard exertion (permutation test, *p* < 0. 0115), hard vs. somewhat hard (permutation test, *p* < 0.0434), hard vs. extremely light (permutation test, *p* < 0.0277), and somewhat hard vs. extremely light exertion levels (permutation test, *p* < 0.0426). However, there was a significant reduction between somewhat hard and light (permutation test, *p* < 0.0247). The extremely hard and hard exertion levels provoked more densely connected neighboring nodes within the network than the light and extremely light exertion levels, as evidenced by the local efficiency shown in (Figure 13d).

#### 4.5.2. Topological Differences in the Global Network for Beta Coherence Network

**Figure 14:**
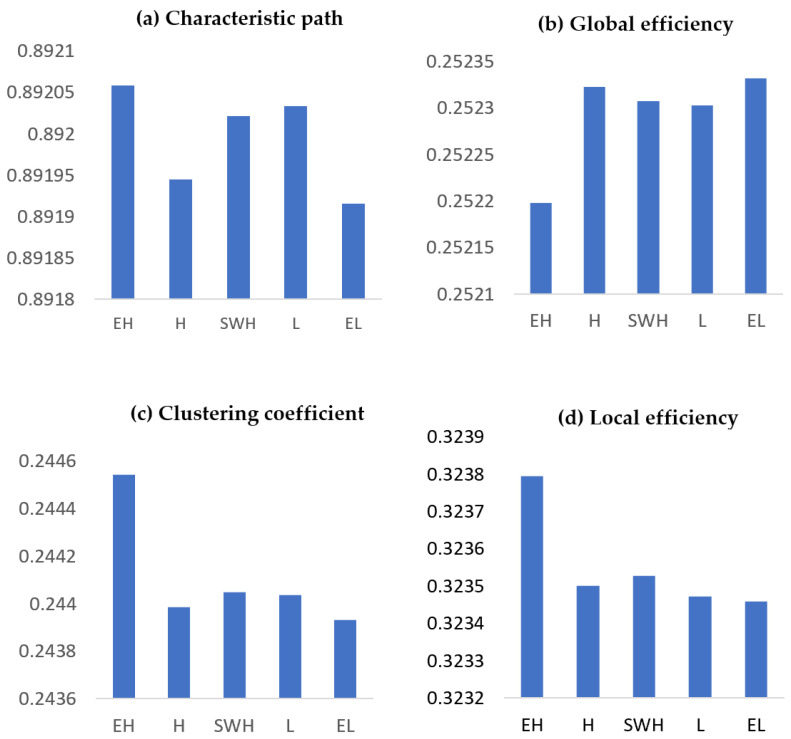
Graph theoretical network metrics showing the primary effects of various exertion levels. (**a**) Characteristic path length. (**b**) Global efficiency. (**c**) Clustering coefficient. (**d**) Local efficiency for beta coherence. EL: extremely light, L: light, SWH: somewhat hard, H: hard, and EH: extremely hard.

The permutation test for global measures yielded significant differences in beta coherence among the various exertion levels. A significant small-world network was exhibited for extremely hard exertion vs. somewhat hard (permutation test, *p* < 0.0396), somewhat hard vs. hard (permutation test, *p* < 0.0042), somewhat hard vs. both light (permutation test, *p* < 0.0385) and extremely light (permutation test, *p* < 0.001), and extremely light vs. light exertion levels (permutation test, *p* < 0.0309). Maximum values for both the characteristic path length and clustering coefficient were observed at extremely hard exertion, whereas minimum values were found at the extremely light exertion level (Figure 14a). A significant reduction in characteristic path length was observed for extremely hard vs. hard exertion (permutation test, *p* < 0.0239), extremely hard vs. light (permutation test, *p* < 0.00), and extremely hard vs. extremely light (permutation test, *p* < 0.0027). There were significant declines between hard and light (permutation test, *p* < 0.00) and between light and extremely light (permutation test, *p* < 0.00). However, a significant increase in characteristic path length was found for hard compared with both somewhat hard (permutation test, *p* < 0.0171) and light (permutation test, *p* < 0.00). Overall, there was a significant increase in global efficiency for the lower exertion levels compared with higher exertion levels (Figure 14a). A sharp decline in the clustering coefficient was observed for extremely light exertion compared with the other exertion levels (Figure 14c). Consequently, significant reductions in local efficiency were observed for the extremely light exertion level compared with the other exertion levels (Figure 14d).

#### 4.5.3. Topological Differences in the Local Network

##### Betweenness Centrality Results

For all exertion levels, the key nodes were located in the left superior frontal (BA 10), right inferior frontal (BAs 45 and 47), left precuneus (BAs 7 and 31), right middle temporal (BA 21), left fusiform gyrus (BA 37), left superior temporal (BA 41), and right lingual gyrus (BA 17) regions for the alpha band. For the beta network, significant differences were observed only in the inferior frontal region (BA 47) for all force exertion levels. The key node with the highest betweenness centrality for the alpha network at the extremely hard exertion level was located in the superior frontal gyrus in the right frontal lobe, corresponding to BA 10. For all other exertion levels, the key node with the highest betweenness centrality was found in the left superior frontal brain region, corresponding to BA 11. The key node with the highest betweenness centrality in the beta network for all exertion levels was located in the left lingual gyrus in the occipital lobe (BA 17).

##### Degree Centrality Results

**Figure 15:**
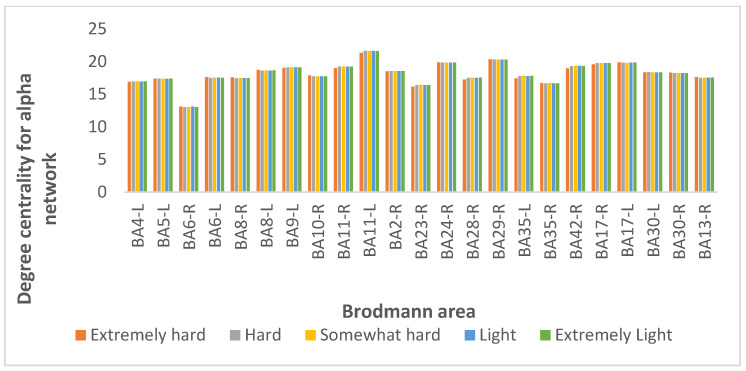
Results for degree centrality for the alpha coherence network at exertion levels extremely hard (orange), hard (gray), somewhat hard (yellow), light (blue), and extremely light (green).

From 84 BAs, we selected the 30% with the highest degree centrality values for all subjects at all exertion levels for the alpha coherence, as shown in Figure 15. The extremely light exertion level exhibited a higher degree of centrality in all network nodes than the other exertion levels. For all exertion levels, the superior frontal gyrus in the orbitofrontal region, corresponding to (BA 11-left), was found to be the most important node in the alpha network in terms of the number of edges incident upon a node.

**Figure 16:**
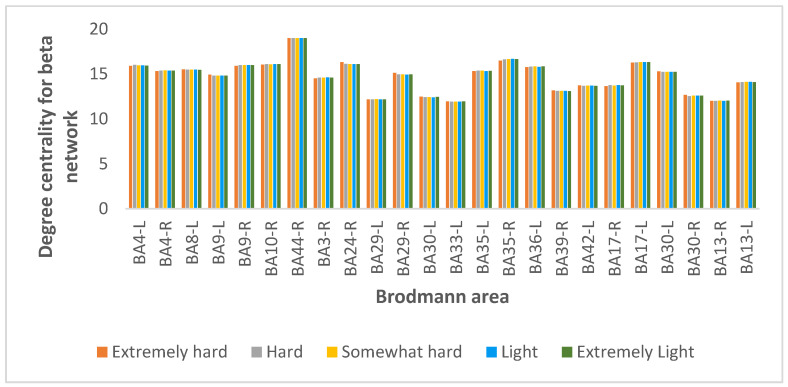
Results for degree centrality for the beta coherence network at exertion levels extremely hard (orange), hard (gray), somewhat hard (yellow), light (blue), and extremely light (green).

From 84 BAs, we selected the 30% with the highest degree centrality values for all subjects at all exertion levels for the beta coherence, as shown in Figure 16. For all exertion levels, the precentral gyrus region of the frontal lobe, corresponding to BA 44, was the most important node in the beta network in terms of the number of edges incident upon a node.

##### Nodal Efficiency

For all exertion levels in the alpha band, the highest regional efficiencies were found in the middle frontal gyrus of the frontal lobe, corresponding to BA 11, and the posterior cingulate of the limbic lobe, corresponding to BA 29. The lowest regional efficiencies were found in the superior frontal of the frontal lobe (BA 10), the inferior frontal region of the frontal lobe (BAs 45 and 47), the precuneus of the parietal lobe (BAs 7 and 31), the middle temporal gyrus of the temporal lobe (BA 21), the fusiform gyrus of the temporal lobe (BA 37), and the lingual gyrus of the occipital lobe (BA 17). Interestingly, for the beta network, the highest nodal efficiency was found in the precentral gyrus of the frontal lobe (BA 44) and the lingual gyrus of the occipital lobe (BA 17). The minimum nodal efficiency was present in the inferior frontal gyrus of the frontal lobe (BA 47) and the middle temporal gyrus of the temporal lobe (BA 21). A summary of the highest nodal centralities for the alpha and beta bands for each exertion level is given in Table 10.

## 5. Discussion

To the best of our knowledge, this report describes the first task-based EEG study investigating the effect of force exertion on the EEG functional brain network at the source level for healthy female participants using a graph theoretical approach. We demonstrated that graph theoretical measures applied to source EEG data could be used to identify brain network topological properties induced by different force exertion levels. First, we established an EEG pre-processing flow chart to construct the EEG functional brain network at the source level. Second, using the coherence method, we computed the functional connectivity patterns induced by various force exertion levels at different frequency bands. Finally, we computed global and local graph theoretical measures to characterize the functional brain network for each exertion level and frequency band.

We obtained findings concerning (a) force measures and RPPC scores, (b) functional brain patterns, and (c) global and local graph theoretical measures for both frequency measures.

### 5.1. Force Measures and RPPC Scores

Our study revealed no significant differences in exerted forces between hard vs. somewhat hard or light vs. extremely light levels. As expected, a negative correlation between the RPPC and exerted force was also found. These results suggest that, as the level of exerted force in a physical task increases, the participant’s feeling of task comfort declines. This observation sheds light on the effect of perceived physical comfort on neural activity.

### 5.2. Functional Brain Patterns

Functional connectivity estimators were computed using the coherence method, which has been shown to be sufficient to capture the amount of shared activity between brain regions in the frequency domain [15,119,120,121,122,123,124].

The alpha network demonstrated stronger coupling in the frontoparietal brain regions at the highest exertion level compared with all other exertion levels. It should be noted that the frontoparietal alpha network reflects attention modulation and perceptual regulation [142]. Furthermore, increments in functional connectivity over the frontoparietal lobe may indicate the progression of muscular fatigue [143]. In detail, when the exertion level increases, there is a strong coupling between the left paracentral (BA 5) and left lingual gyrus (BA 17) in the alpha band. The BA 5 region is involved in somatosensory processing, motor control, and association [144], whereas BA 17 is involved in discerning the intensity of an object (i.e., primary visual cortex). This study identified a strong coupling between the left superior frontal gyrus (BA 10) and the middle frontal gyrus (BA 11). In general, BA 10 is involved in various executive brain functions, whereas BA 11 involves planning, decision-making, and reward processing. Disconnections were found between the middle frontal gyrus (BA 14) and the anterior cingulate (BA 33) as the exertion level increased. Because the anterior cingulate plays an important role in cognitive control, emotions [145], working memory processing, and decision-making, such disconnections might indicate impairment in cognitive performance leading to a deterioration in the task response time [146,147,148,149].

The beta network was found to reflect strong coupling in the right superior frontal gyrus and parahippocampal gyrus for the highest exertion level compared with all other levels. These findings suggest that physical activity may be associated with larger brain gray matter volume [150], as physical activity is associated with the hippocampus and prefrontal cortex [151,152].

### 5.3. Brain Network

Using graph theoretical measures, we investigated global and local alterations in the cortical functional connectivity network in the alpha and beta bands for all predefined force exertion levels.

#### 5.3.1. Global Measures

A higher clustering coefficient for alpha and beta coherence was observed for extremely hard vs. extremely low exertion. This observation suggests an increase in the functional segregation of the brain network during high-force exertions. An increase in the clustering coefficient during an isometric finger movement task was previously reported by Storti et al. [153], indicating a strong connection between neighbor nodes in the network during a voluntary arm movement task. However, high mental workload tasks have been found to reduce local clustered connectivity [154]. Others have suggested that increases in the clustering coefficient are associated with better working memory performance [37].

The reduction in characteristic path length at high exertion levels in the alpha network may reflect a higher global efficiency for transferring parallel information. Therefore, we postulate that the brain is more efficient in processing and transferring information when a physical task requires more exertion. These results align with previous studies [155,156,157,158]. Furthermore, the exhibition of small-world organization for the alpha coherence network might indicate a high level of local segregation and globally integrated networks under extremely hard vs. extremely low exertion levels. These results are also consistent with a study by Ren et al. [46], who found an increase in small-worldness in the alpha band during the performance of a task with a high workload level compared with an easy task. We found that the functional brain network shifted to a more ordered configuration for the beta network. Similar phenomena have been observed in brain activity after a sustained-attention task [159].

The global efficiency increased during the hard exertion condition for the alpha band but not for the beta band. This finding suggests enhanced performance during the hard exertion task, with a higher level of processing integration in the brain network. The higher global structure in the alpha band might be attributed to the importance of the alpha network in information processing, the cognitive domain, and the need for certain types of attention for coping with high-force tasks [160].

Greater cognitive efforts induce the presence of human functional brain networks that are more efficient but also exhibit less economical network configurations [161]. Furthermore, mentally fatiguing tasks have been associated with human functional brain networks that are more economical but also less efficient [45,162]. In this study, an increase in local efficiency for both frequency bands was associated with elevated force exertion levels. In accord with previous findings [37,163,164], the increment in local efficiency suggests that brain regions communicate and cooperate to a larger degree as the physical force exertion level increases. Finally, the results of this study suggest that both alpha and beta networks exhibit dense connections between nodes within modules but sparse connections between nodes in different modules, thus indicating that the brain is more segregated at high levels of exertion.

Modularity is a good estimator of network robustness [165] and has been used to predict changes in working memory capacity [166]. The results of the present study suggest that high-force tasks provoke alpha coherence networks with a more modular network configuration, contrary to previously reported results regarding cognitive effort effects [161].

#### 5.3.2. Local Measures

Densely connected nodes strongly affect the functional integration and segregation of brain organization, causing a loss of network flexibility when damaged. Three centrality measures were calculated for 84 ROIs, including the betweenness and degree centrality and nodal efficiency, to investigate the effects of force exertion levels on nodal properties. The nodes with the highest betweenness centrality are known as highly central nodes or hubs. Such a node might play a controlling role in the passage of information through the network. The key node with the highest betweenness centrality for the extremely hard exertion level in the alpha network was located in the superior frontal gyrus in the right frontal lobe, corresponding to BA 10. These findings suggest that this brain region is critical for efficient information processing within the brain network for tasks that require force exertions. In contrast, for all other exertion levels, the key node with the highest betweenness centrality was located in the left superior frontal, corresponding to BA 11. For the beta network, the key node with the highest betweenness centrality for all exertion levels was located in the left lingual gyrus of the occipital lobe (BA 17). Therefore, we suggest that these brain regions are critical for efficient information processing within the brain network for physical force exertion tasks.

In terms of the number of edges incident upon a node, a high degree of centrality was found to be associated with the extremely light exertion level in the brain network. For all exertion levels, the superior frontal gyrus in the orbitofrontal region (BA 11) was found to be the most important node in the alpha network, and the precentral gyrus region of the frontal lobe (BA 44) was the most important node in the beta network.

The nodal efficiency results suggest that the superior frontal gyrus acts as a hub for the alpha network at all exertion levels, whereas both the precentral and parahippocampal gyri act as hub nodes for the beta network at all exertion levels.

Finally, we provided a comparative table that summarizes and compares the proposed method’s main virtues versus the state-of-the-art report in Table 11. The main virtues include the novelty of studying the perception of force exertion using graph theory measures obtained from brain data, using different global and local measures to understand the topological properties of the functional brain network, focusing on female subjects only, and considering the EEG data from the source level.

## 6. Limitations and Future Implications

The results of the present study demonstrate that graph theoretical measures can be used to quantify changes in brain network topological properties induced by various force exertions. However, many challenges must still be addressed to achieve further progress. Although the existing literature suggests that the sample size of 12 participants is not too small compared to other studies [18,143,172,173,174,175,176,177,178], a larger sample size would be valuable for increasing the power of the study. More subjects should be recruited to validate the proposed analysis method further. We believe the current findings in this first study that applied a graph theoretical approach to model functional brain connectivity might provide evidence and new insights into neuroergonomics applications. We used a nonparametric comparison to reduce the effect of the possible non-normality in some variables caused by the limited sample size. We also addressed the potential limitations of EEG-based neuroergonomics studies focusing only on male participants [64]. Future studies will include male participants only to compare the results obtained from females since sex is an important biological variable in brain research. The majority of previous works binarized the brain network to remove weak, noisy, and insignificant connections in the network; others reported that weighted graphs may contain more information and might ensure greater sensitivity in response to distractor effects compared with unweighted graphs [122,179]. In particular, the choice of thresholding value is crucial because this value significantly affects the network topology properties; no threshold values are free from bias, suggesting the need for future investigation [180,181]. The superior temporal resolution of EEG helps to capture dynamic changes in brain activity underlying critical aspects of cognition and behavior. Consequently, implementing a dynamic functional connectivity method is promising for future neuroergonomics studies [182]. Future research may consider a new method to reduce the special variance resulting from the EEG’s poor spatial resolution. One novel approach is based on the spectral correlation with a Movement Related Independent Component to sort the estimated sources by Blind Source Separation [83]. Future research is needed to study the perception of static and dynamic force exertions in other body regions, such as the legs and torso.

Future studies should also consider the neuromuscular coupling analysis to reflect the interaction between the cerebral motor cortex and affected muscles [172].

## 7. Conclusions

The present study has demonstrated that a graph theory approach incorporating coherence helps characterize the frequency-specific neurophysiological bases of perceived exertion. Our finding based on coherence estimation demonstrated a stronger coupling in the frontoparietal alpha network at the highest exertion level compared with all other exertion levels reflecting a high level of attention modulation and perceptual regulation. Additionally, the beta network demonstrated a strong coupling in the right superior frontal gyrus and parahippocampal gyrus for the highest exertion level compared with all other levels. Our findings, based on graph theoretical measures, show that “extremely hard” force exertions provoked alpha networks with a greater clustering coefficient, more modularity, higher local efficiency, and higher global efficiency, suggesting that brain regions communicate and cooperate to a larger degree as the physical force exertion level increases. However, we found a lower global efficiency for the beta network, which might indicate a reduction in cognitive impairment [183]. The orbitofrontal region of the superior frontal gyrus of the brain was found to be the most critical node in the alpha network based on the calculations of betweenness and degree centrality. The applied global and local graph theoretical measures characterized functional segregation and integration for the brain network. The application of network modulation allowed us to study the functional brain network properties of different brain regions during arm exertion tasks, with the aim of increasing our current understanding of brain function during physical task performance. The results of this study appear to provide additional evidence confirming the notion that the human brain reorganizes and recruits more resources to efficiently respond to increasing physical task demands in terms of exerted forces in addition to maintaining a required level of task attention [154,164,184].

## Figures and Tables

**Table 1:** Descriptive statistics of anthropometric measurements and maximum voluntary contraction (MVC) measured in newton(N). SD: standard deviation.

Variable	Mean	SD
Age (year)	27.4	6.2
Body Weight (kg)	60.2	11
Shoulder Height (cm)	135.85	7.50
Hip Height (cm)	98.04	6.08
Knee Height (cm)	51.65	2.84
Arm Height (cm)	106.26	5.90
Knuckle Height (cm)	73.98	6.28
Body Height (cm)	163.00	7.260
MVC (N)	115.00	47.60

**Table 2:** Statistics of isometric arm exertion forces, including the mean, standard deviation (SD), range, and percentage of maximum voluntary contraction (MVC) at different physical exertion levels.

Predefined Level of Physical Exertion	Isometric Arm Force (N)						
	Mean	SD	Range		% MVC		
			Min	Max	Mean	Min	Max
Extremely hard	67.35	35.25	2	18	49%	3%	99%
Hard	41.83	18.9	3	28	30%	4%	60%
Somewhat hard	34.58	16.7	3	66	25%	2%	48%
Light	13.61	6.76	6	82	10%	2%	20%
Extremely light	8.04	5.32	4	136	4%	1%	13%

**Table 3:** ANOVA results for the effect of exertion level on the exerted arm force (N). D: degree of freedom, MS: mean sum of squares, and SS: sum of squares.

Source	Df	Adj SS	Adj MS	F-Value	*p*-Value
Participant	11	11,374	1034.0	4.05	0.00
Exertion level	4	27,108	6777.085	26.54	0.00
Error	44	11,236	255.4		
Total	59	49,718			

**Table 4:** Summary statistics for arm forces exerted at different levels of physical exertion (Tukey pairwise comparison at the 95% confidence level).

Exertion Level	Mean	Grouping
Extremely hard	67.4	A
Hard	41.83	B
Somewhat hard	34.57	B
Light	13.62	C
Extremely light	8.04	C

**Table 5:** Results of Tukey’s post hoc simultaneous tests for differences in means for forces exerted at different physical exertion levels. EL: extremely light, L: light, SWH: somewhat hard, H: hard, EH: extremely hard, CI: confidence interval, NS: not significant, and SE: standard error.

Difference in Exertion Level	Difference in Mean	SE of Difference	Simultaneous 95% CI	T-Value	Adj *p*-Value
EL-EH	−59.31	6.52	(−77.85, −40.76)	−9.09	0.000
H-EH	−25.52	6.52	(−44.06, −6.97)	−3.91	0.003
L-EH	−53.73	6.52	(−72.28, −35.19)	−8.24	0.000
SWH-EH	−32.77	6.52	(−51.32, −14.23)	−5.02	0.000
H-EL	33.79	6.52	(15.25, 52.34)	5.18	0.000
L-EL	5.58	6.52	(−12.97, 24.12)	0.85	NS
SWH-EL	26.53	6.52	(7.99, 45.08)	4.07	0.002
L-H	−28.22	6.52	(−46.76, −9.67)	−4.33	0.001
SWH-H	−7.26	6.52	(−25.80, 11.29)	−1.11	NS
SWH-L	20.96	6.52	(2.41, 39.50)	3.21	0.020

**Table 6:** Mean and standard deviation for the rate of perceived physical comfort at each exertion level.

Exertion Level	RPPC Mean	RPPC Standard Deviation
Extremely light	8.80	1.48
Light	8.16	1.63
Somewhat hard	5.47	1.68
Hard	5	2.11
Extremely hard	3.9	2.30

**Table 7:** ANOVA results for the effect of exertion level on the rate of perceived physical comfort scores. DF: degree of freedom, MS: mean sum of squares, and SS: sum of squares.

Source	Df	Adj SS	Adj MS	F-Value	*p*-Value
Participants	11	61.71	5.60	7.56	0.000
Exertion levels	4	119.36	29.84	40.23	0.000
Errors	44	32.64	0.7417		
Total	59	213.71			

**Table 8:** Summary statistics for the rate of perceived physical comfort scores at various levels of physical exertion (Tukey pairwise comparison at the 95% confidence level).

Exertion Level	Mean	Grouping
Extremely hard	4.583	A
Hard	5.375	AB
Somewhat hard	5.729	B
Light	7.750	C
Extremely light	8.23	C

**Table 9:** Results of Tukey’s simultaneous tests for differences in the rate of perceived physical comfort scores at various levels of physical exertion. NS: not significant.

Difference in Exertion Level	Difference in Mean	SE of Difference	Simultaneous 95% CI	T-Value	Adj *p*-Value
EL-EH	3.646	0.352	(2.646, 4.645)	10.37	0.000
H-EH	0.792	0.352	(−0.208, 1.791)	2.25	0.180
L-EH	3.167	0.352	(2.167, 4.166)	9.01	0.000
SWH-EH	1.146	0.352	(0.146, 2.145)	3.26	0.017
H-EL	−2.854	0.352	(−3.854, −1.855)	−8.12	0.000
L-EL	−0.479	0.352	(−1.479, 0.520)	−1.36	NS
SWH-EL	−2.500	0.352	(−3.499, −1.501)	−7.11	0.000
L-H	2.375	0.352	(1.376, 3.374)	6.75	0.000
SWH-H	0.354	0.352	(−0.645, 1.354)	1.01	NS
SWH-L	−2.021	0.352	(−3.020, −1.021)	−5.75	0.000

**Table 10:** Summary of the highest nodal centrality for the alpha and beta networks for each exertion level.

Nodal Centrality	Frequency Band	Extremely Hard	Hard	Somewhat Hard	Light	Extremely Light
Betweenness centrality	Alpha	BA 10	BA 11	BA 11	BA 11	BA 11
Betweenness centrality	Beta	BA 17	BA 17	BA 17	BA 17	BA 17
Degree centrality	Alpha	BA 11	BA 11	BA 11	BA 11	BA 11
Degree centrality	Beta	BA 44	BA 44	BA 44	BA 44	BA 44
Nodal efficiency	Alpha	BA 11, 29	BA 11, 29	BA 11, 29	BA 11, 29	BA 11, 29
Nodal efficiency	Beta	BA 44	BA 44	BA 44	BA 44	BA 44

**Table 11:** Comparison of the proposed method’s main virtues vs. the state-of-the-art report. Bw: Betweenness centrality, PL: Characteristic path length, CC: Clustering coefficient, D: Degree, Eg: Global efficiency, Eloc: Local efficiency, Enodal: Efficiency of nodal, N/A: Not applicable, and SW: Small-world organization.

Reference	Task	Global Measures	Local Measures	Number of Participants	ROI	Frequency Bands
Our study	Isometric arm exertion	Eg, El, CC, PL, SW	Bw, D, Enodal	12 females	84 sources based	Alpha and beta
Fallani et al. [167]	Foot dorsal flexion	Eg, Eloc	N/A	5	16 sources based	Alpha
Jin et al. [168]	Finger-tapping	Eg	Enodal	12 males 3 females	Scalp based	Alpha and beta
Kar et al. [169]	Physical activity	CC, PL	D	12 males	Scalp based	Alpha and theta
Sengupta et al. [170]	Physical exercise	CC, PL	N/A	12 males	Scalp based	N/A
Storti et al. [171]	Arm movement	CC	Dc	7 males 3 females	Scalp based	Alpha and beta
Storti et al. [153]	Reach and grasp	Weighted CC, weighted PL	N/A	10	Scalp based	Alpha, beta, delta, and theta
Huang et al. [163]	Play task	Eg, Eloc	D	19 males	Scalp based	Beta and theta
Porter et al. [17]	Cycling	CC	N/A	8 males 5 females	Scalp based	Theta

## Data Availability

The data presented in this study are available upon request from the corresponding author.

## Supplementary Materials

The following supporting information can be downloaded at: https://www.mdpi.com/article/10.3390/brainsci12111575/s1, Supplementary Material A: Montreal Neurophysiological Institute (MNI) coordinates of the 84 regions of interest (ROI) used to analyze the electroencephalograph signal of each exertion level.
