# Supplementary material for: Modeling Brain Functional Connectivity Patterns during an Isometric Arm Force Exertion Task at Different Levels of Perceived Exertion: A Graph Theoretical Approach
Source: Brain Sci. 2022 Nov 18;12(11):1575. doi: 10.3390/brainsci12111575 (PMC9688629; doi:10.3390/brainsci12111575)
Supplement: Supplementary file 1 [file brainsci-12-01575-s001.zip › brainsci-2007752-supplementary.pdf]

Supplementary Material A

Montreal Neurophysiological Institute (MNI) coordinates of the 84 regions of interest (ROI) used to analyze the electroencephalograph signal of each exertion level.

Hemisphere	MNI X	MNI Y	MNI Z	Lobe	Structure	Brodmann area	ROI
Left	−35	−25	55	Frontal Lobe	Precentral Gyrus	BA 4	1
Right	35	−20	50	Frontal Lobe	Precentral Gyrus	BA 4	2
Right	15	−45	60	Frontal Lobe	Paracentral Lobule	BA 5	3
Left	−15	−45	60	Frontal Lobe	Paracentral Lobule	BA 5	4
Right	30	−5	55	Frontal Lobe	Middle Frontal Gyrus	BA 6	5
Left	−30	−5	55	Frontal Lobe	Middle Frontal Gyrus	BA 6	6
Right	20	25	50	Frontal Lobe	Superior Frontal Gyrus	BA 8	7
Left	−20	30	50	Frontal Lobe	Superior Frontal Gyrus	BA 8	8
Left	−30	30	35	Frontal Lobe	Middle Frontal Gyrus	BA 9	9
Right	30	30	35	Frontal Lobe	Middle Frontal Gyrus	BA 9	10
Right	25	55	5	Frontal Lobe	Superior Frontal Gyrus	BA 10	11
Left	−25	55	5	Frontal Lobe	Superior Frontal Gyrus	BA 10	12
Right	20	45	−20	Frontal Lobe	Superior Frontal Gyrus	BA 11	13
Left	−20	40	−15	Frontal Lobe	Middle Frontal Gyrus	BA 11	14
Right	5	15	−15	Frontal Lobe	Subcallosal Gyrus	BA 25	15
Left	−10	20	−15	Frontal Lobe	Medial Frontal Gyrus	BA 25	16
Right	55	10	15	Frontal Lobe	Precentral Gyrus	BA 44	17
Left	−50	10	15	Frontal Lobe	Precentral Gyrus	BA 44	18
Right	50	20	15	Frontal Lobe	Inferior Frontal Gyrus	BA 45	19
Left	−50	20	15	Frontal Lobe	Inferior Frontal Gyrus	BA 45	20
Right	45	35	20	Frontal Lobe	Middle Frontal Gyrus	BA 46	21
Left	−45	35	20	Frontal Lobe	Middle Frontal Gyrus	BA 46	22
Right	30	25	−15	Frontal Lobe	Inferior Frontal Gyrus	BA 47	23
Left	−30	25	−15	Frontal Lobe	Inferior Frontal Gyrus	BA 47	24
Left	−55	−25	50	Parietal Lobe	Postcentral Gyrus	BA 2	25
Left	−45	−30	45	Parietal Lobe	Postcentral Gyrus	BA 2	26
Right	55	−25	50	Parietal Lobe	Postcentral Gyrus	BA 2	27
Right	35	−25	50	Parietal Lobe	Postcentral Gyrus	BA 3	28
Right	40	−25	50	Parietal Lobe	Postcentral Gyrus	BA 3	29
Left	−20	−65	50	Parietal Lobe	Precuneus	BA 7	30
Right	15	−65	50	Parietal Lobe	Precuneus	BA 7	31
Left	−10	−50	30	Parietal Lobe	Precuneus	BA 31	32
Right	10	−50	35	Parietal Lobe	Precuneus	BA 31	33

Right	50	−30	45	Parietal Lobe	Inferior Parietal Lobule	BA 40	34
Right	50	−45	45	Parietal Lobe	Inferior Parietal Lobule	BA 40	35
Left	−50	−40	40	Parietal Lobe	Inferior Parietal Lobule	BA 40	36
Left	−5	−40	25	Limbic Lobe	Posterior Cingulate	BA 23	37
Right	5	−45	25	Limbic Lobe	Posterior Cingulate	BA 23	38
Right	5	0	35	Limbic Lobe	Cingulate Gyrus	BA 24	39
Right	5	30	20	Limbic Lobe	Anterior Cingulate	BA 24	40
Left	−5	0	35	Limbic Lobe	Cingulate Gyrus	BA 24	41
Left	−5	30	20	Limbic Lobe	Anterior Cingulate	BA 24	42
Right	20	−35	−5	Limbic Lobe	Parahippocampal Gyrus	BA 27	43
Left	−20	−35	−5	Limbic Lobe	Parahippocampal Gyrus	BA 27	44
Left	−20	−10	−25	Limbic Lobe	Parahippocampal Gyrus	BA 28	45
Right	20	−10	−25	Limbic Lobe	Parahippocampal Gyrus	BA 28	46
Left	−5	−50	5	Limbic Lobe	Posterior Cingulate	BA 29	47
Right	5	−50	5	Limbic Lobe	Posterior Cingulate	BA 29	48
Left	−15	−60	5	Limbic Lobe	Posterior Cingulate	BA 30	49
Left	−5	20	20	Limbic Lobe	Anterior Cingulate	BA 33	50
Right	0	20	20	Limbic Lobe	Anterior Cingulate	BA 33	51
Right	15	0	−20	Limbic Lobe	Parahippocampal Gyrus	BA 34	52
Left	−15	0	−20	Limbic Lobe	Parahippocampal Gyrus	BA 34	53
Left	−20	−25	−20	Limbic Lobe	Parahippocampal Gyrus	BA 35	54
Right	30	−25	−25	Limbic Lobe	Parahippocampal Gyrus	BA 35	55
Right	25	−25	−20	Limbic Lobe	Parahippocampal Gyrus	BA 35	56
Left	−30	−30	−25	Limbic Lobe	Parahippocampal Gyrus	BA 36	57
Right	−5	−40	25	Limbic Lobe	Posterior Cingulate	BA 23	58
Left	−45	−20	−30	Temporal Lobe	Fusiform Gyrus	BA 20	59
Left	−60	−20	−15	Temporal Lobe	Middle Temporal Gyrus	BA 21	60
Right	60	−15	−15	Temporal Lobe	Middle Temporal Gyrus	BA 21	61
Left	−45	−55	−15	Temporal Lobe	Fusiform Gyrus	BA 37	62
Right	45	−55	−15	Temporal Lobe	Fusiform Gyrus	BA 37	63
Left	−40	15	−30	Temporal Lobe	Superior Temporal Gyrus	BA 38	64
Right	40	15	−30	Temporal Lobe	Superior Temporal Gyrus	BA 38	65
Right	45	−65	25	Temporal	Middle Temporal Gyrus	BA 39	66

				Lobe			
Left	−45	−65	25	Temporal Lobe	Middle Temporal Gyrus	BA 39	67
Left	−45	−30	10	Temporal Lobe	Transverse Temporal Gyrus	BA 41	68
Right	55	−20	5	Temporal Lobe	Superior Temporal Gyrus	BA 41	69
Left	−55	−25	5	Temporal Lobe	Superior Temporal Gyrus	BA 41	70
Right	45	−30	10	Temporal Lobe	Transverse Temporal Gyrus	BA 41	71
Left	−60	−10	15	Temporal Lobe	Transverse Temporal Gyrus	BA 42	72
Left	−60	−25	10	Temporal Lobe	Superior Temporal Gyrus	BA 42	73
Right	60	−10	15	Temporal Lobe	Transverse Temporal Gyrus	BA 42	74
Right	65	−25	10	Temporal Lobe	Superior Temporal Gyrus	BA 42	75
Right	15	−85	0	Occipital Lobe	Lingual Gyrus	BA 17	76
Right	10	−90	0	Occipital Lobe	Lingual Gyrus	BA 17	77
Left	−10	−90	0	Occipital Lobe	Lingual Gyrus	BA 17	78
Left	−15	−85	0	Occipital Lobe	Lingual Gyrus	BA 17	79
Left	−25	−75	10	Occipital Lobe	Cuneus	BA 30	80
Right	10	−60	5	Occipital Lobe	Cuneus	BA 30	81
Right	25	−75	10	Occipital Lobe	Cuneus	BA 30	82
Right	40	−5	10	Sub-lobar	Insula	BA 13	83
Left	−40	−10	10	Sub-lobar	Insula	BA 13	84
